# Survey proportion of safety and ergonomics parameters in Sabzevar preschool centers 

**Published:** 2019-08

**Authors:** Fateme Abareshi, Zahra Sharifi, Reza Hekmatshoar

**Affiliations:** ^*a*^Sabzevar University of Medical Sciences, Sabzevar, Iran.

## Abstract

**Background:**

The first months and years of life of the child play the most important role in the formation and growth of his mental, physical and mental dimensions. Children, on the other hand, are one of the most vulnerable groups of the community, however, children need to play in order to understand their surroundings. This means that design for children should be in a way that balances their motivation and safety. This study was conducted to evaluate the fit of ergonomic indices in preschool centers in Sabzevar.

**Methods:**

This descriptive cross-sectional study was conducted among preschools centers under the supervision of the Welfare Organization in Sabzevar city. Only 39 center from 50 center tended to participate in this study. Ergonomics and safety indices in two parts of the physical and cognitive part were measured using a researcher made checklist was designed and validated by Aziz-Dabadi Farahani. Data was gathered and then analyzed using SPSS.16 software.

**Results:**

The results of this study showed that the preschool centers were considered for each child (6.4%), the height of the wash basin (68.42%), the playing room floor (22.2% 72), wall coverings (87.17%), lighting (49%), temperature (66.3%), window shields (35.89%), fire extinguisher charging (74.41%), The corridor width leading to the playing room (38.88%) was favorable. But the sound level in any of the preschool centers was not favorable.

**Conclusions:**

Preschool centers and kindergarten have a crucial role in providing safety for children as the most vulnerable and curious group in society. Considering that the assessment and inspection of preschool centers is carried out by environmental authorities, but some of the issues related to the field of occupational health are not properly and accurately addressed, so it is advisable that the occupational health professional Attending inspections, as well as due to the lack of stringency to build preschool centers in suitable places, there are not enough criteria that should be taken into consideration.

**Keywords:**

Preschool centers, Safety, Vulnerable group

